# Supplementary material for: Kirkendall effect-induced uniform stress distribution stabilizes nickel-rich layered oxide cathodes
Source: Nat Commun. 2024 Feb 19;15:1503. doi: 10.1038/s41467-024-45373-1 (PMC10876981; doi:10.1038/s41467-024-45373-1)
Supplement: Supplementary file 1 — Supplementary Information [file 41467_2024_45373_MOESM1_ESM.pdf]

Supplementary Information for:

Kirkendall effect induced uniform stress distribution stabilizes high-nickel layered oxide cathodes

Ziyao Gao[1,2], Chenglong Zhao[1,*], Kai Zhou[1,2], Junru Wu[1,2], Yao Tian[1], Xianming Deng[1,2], Lihan Zhang[1,2], Kui Lin[1,2], Feiyu Kang[1], Lele Peng[1,*], Marnix Wagemaker[3,*] and Baohua Li[1,*]

[1]Institute of Materials Research, Tsinghua Shenzhen International Graduate School, Tsinghua University, Shenzhen 518055, China

[2]School of Materials Science and Engineering, Tsinghua University, Beijing 100084, China.

[3]Department of Radiation Science and Technology, Delft University of Technology, Mekelweg 15, 2629JB Delft, the Netherlands.

* Email: c.zhao-1@tudelft. nl; penglele@sz.tsinghua.edu.cn;

m.wagemaker@tudelft.nl; libh@mail.sz.tsinghua.edu.cn

Research Note I

Growth mechanism. The $Ni(OH)_2$ particles prefer to grow on the Al_2O_3 seeds in the heterogeneous nucleation process. The bonding energy across the substrate interface was compared in **Fig. S2**,[1] including Al_2O_3 to Al_2O_3, $xNi(OH)_2$ to $(1-x)Al_2O_3$ and $Ni(OH)_2$ to $Ni(OH)_2$ as shown. The self-bonding energies of Al2O3-Al2O3 and $Ni(OH)_2$-$Ni(OH)_2$ were 0 and 5.3 kJ/mol, respectively. In this work, the molar ratio of Ni to Al was accurately preset at 96:4, and the bonding energy of the corresponding composition points (corresponding to red star in **Fig. S2**) should be larger than those self-bonding energies. When Al_2O_3 oxide seeds are added into the reaction solution, due to the fact that the bonding energy of $xNi(OH)_2$-$(1-x)Al_2O_3$ is stronger than that of $Ni(OH)_2$-$Ni(OH)_2$ (**Fig. S2**), These energy values suggest that on one hand alumina would not agglomerate into larger particle, and on the other hand the $Ni(OH)_2$ precursors has a stronger tendency to grow on the alumina rather than forming the individual $Ni(OH)_2$ particles when Al_2O_3 oxide seeds are added into the reaction solution. This can be verified by the SEM images shown in **Fig. S4**. The growth process of $Ni(OH)_2$ layer on alumina is prior to the spontaneous aggregation of $Ni(OH)_2$ grains.

From the perspective of the nucleation energy. The co-precipitation reaction involves the nucleation of primary grains and the growth of precursors, wherein the nucleation reaction was gradually completed after the diffusion process of transition metal ions in the salt solution from pH 5.4 to 11[2]. The nucleation work of homogeneous nucleation was calculated as follow:

$$\Delta G_N^* = \frac{16\pi\gamma^3}{3(\Delta G_V)^2} \quad \cdots\cdots\cdots\cdots\cdots\cdots (1)$$

Wherein γ represents the surface free energy or surface tension; ΔG_V represents ΔG on a per unit volume basis. ΔG_N^* represents the energy barrier for nucleation.

As for heterogenous nucleation, nucleation processes are catalyzed by a heterogeneity such as an accommodating substrate surface.

$$\Delta G_N^* = \frac{16\pi\gamma^3}{3(\Delta G_V)^2} \cdot f(\theta) \ \ (0 < f(\theta) < 1) \quad \cdots\cdots\cdots\cdots\cdots\cdots (2)$$

The energy barriers for heterogeneous nucleation are much lower than those of homogeneous nucleation. In addition, heterogeneous nucleation is widely

recognized to feature higher nucleation rate and finer grain size[3]. Thus, the nickel hydroxide precursors grown on the Al_2O_3 exhibited a dense packed microparticles with distinct core-shell structure. As a result, aluminum oxide which was not covered by precursor material is not observable on the early stage of co-precipitation reaction.

Research Note II

Kirkendall effect in the calcination process. The hk-precursor shows a distinct core-shell structure with Al_2O_3 seeds located in the core. During the annealing process, the Al atoms will be gradually migrated to the outer surface leaving a gradient doping of Al to the secondary particle with higher concentration of Al at the core region and lower concentration at the outer surface. According to the Arrhenius equation[4], there is an exponential relationship between the diffusion coefficient (D) and temperature, which implies that the thermal motion of the atoms intensifies and the diffusion coefficient rises sharply as temperatures increase.

$$D = D_0 e^{-Q/RT} \qquad \cdots\cdots\cdots\cdots\cdots (3)$$

where R is the gas constant and Q is the activation energy. D_0 is the diffusion constant. Temperature has a strong impact on the diffusion constant. In previous studies, oxide powders have been widely used as dopants.[5,6] Higher Al doping will result in smaller primary particle size than those with lower Al doping[7,8], as verified by **Fig. S7e-j**.

Supplementary Figures

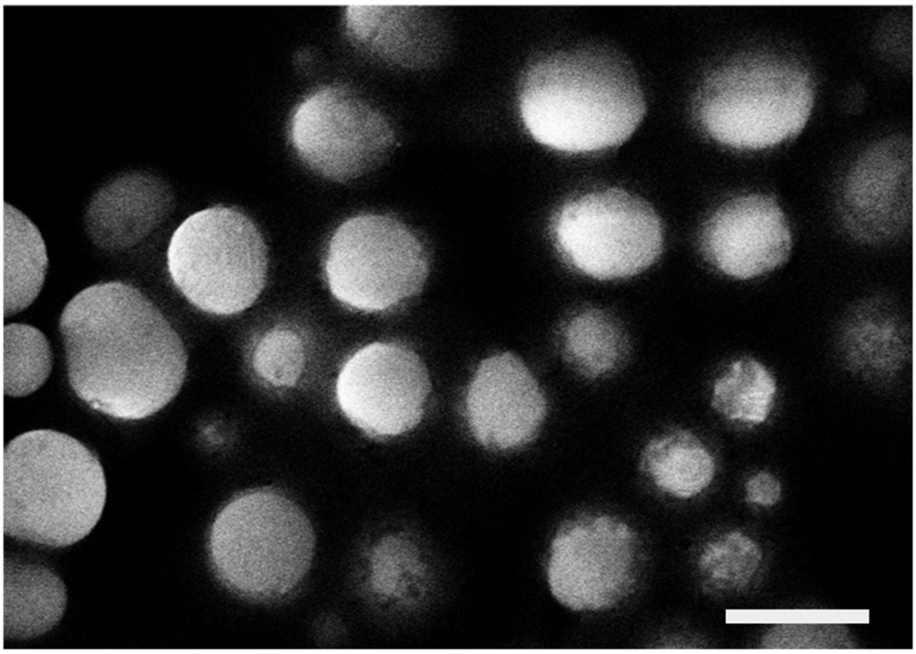

Fig. S1 SEM image of Al_2O_3 seeds. Scale bar, 1 μm.

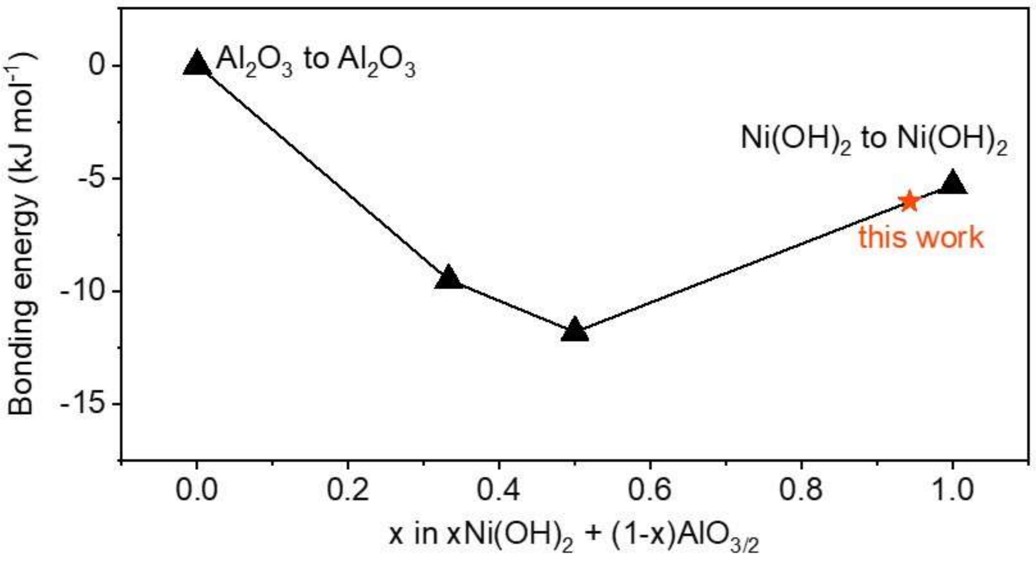

Fig. S2 Bonding energy of Al_2O_3 and $Ni(OH)_2$ as a function of component percentage of $Ni(OH)_2$ in the mixture.

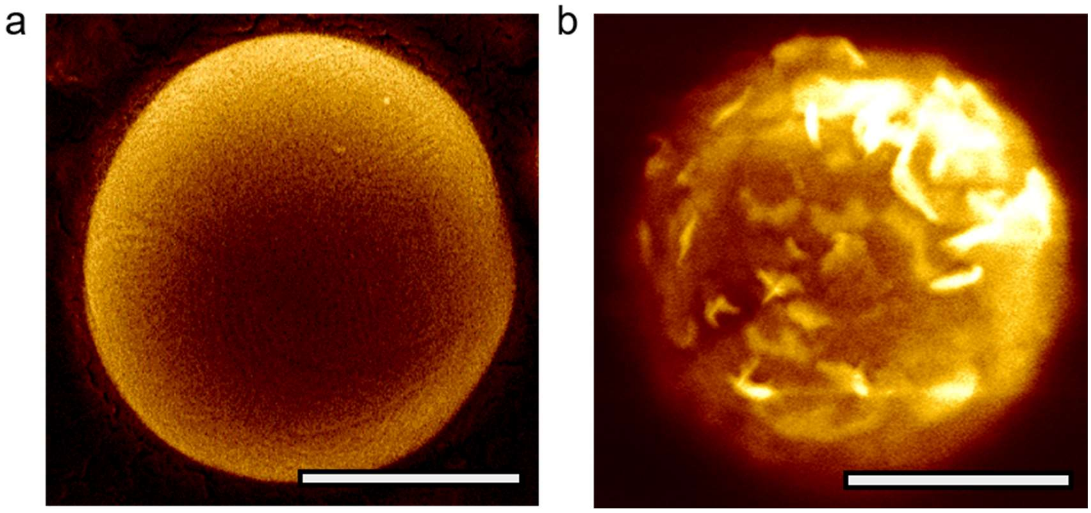

Fig. S3 a SEM images of single Al_2O_3 seed at pristine and **b** during heterogenous nucleation of $Ni(OH)_2$ grains. Scale bar, 500 nm.

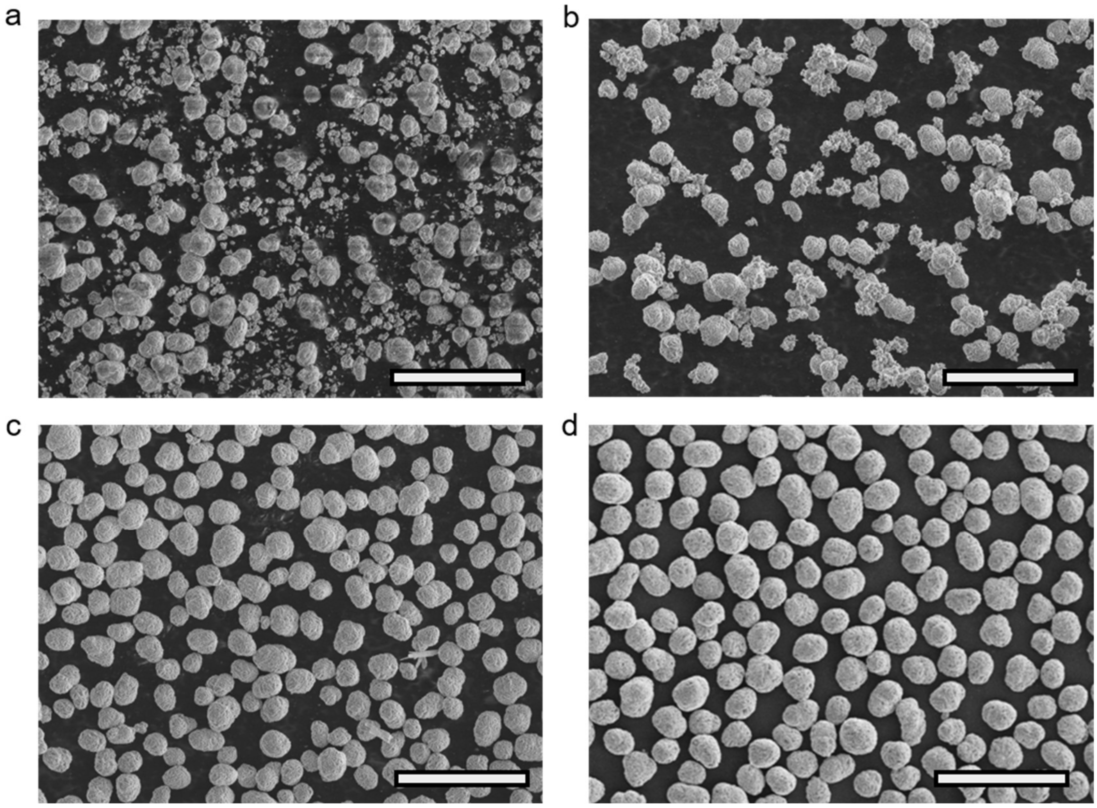

Fig. S4 SEM images of hk-$LiNi_{0.96}Al_{0.04}(OH)_2$ precursors at different reaction times of (**a**) 5 h (**b**) 10 h (**c**) 15h (**d**) 20h during co-precipitation. Scale bar, 30 μm.

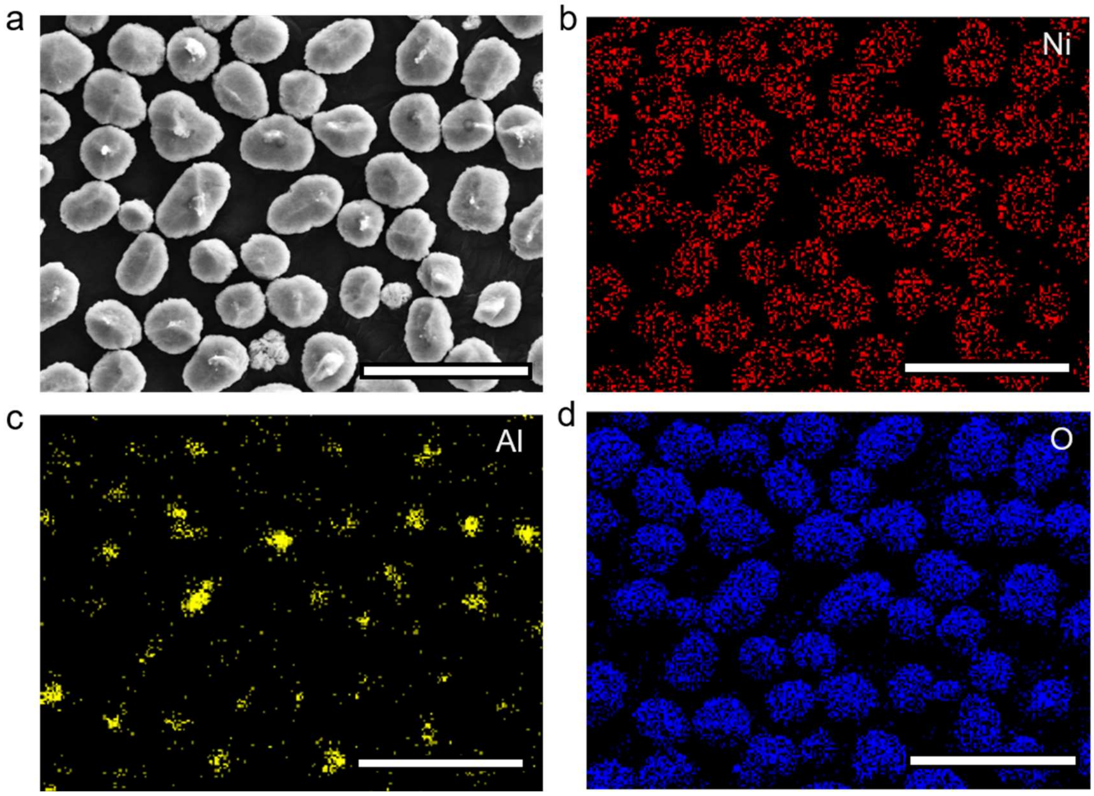

Fig. S5 Distribution of Al and Ni within precursors. a Cross-sectional SEM images and mapping photograph of Ni (**b**), Al (**c**) and O (**d**) within precursor particles for hk-$LiNi_{0.96}Al_{0.04}O_2$. Scale bar, 20 μm.

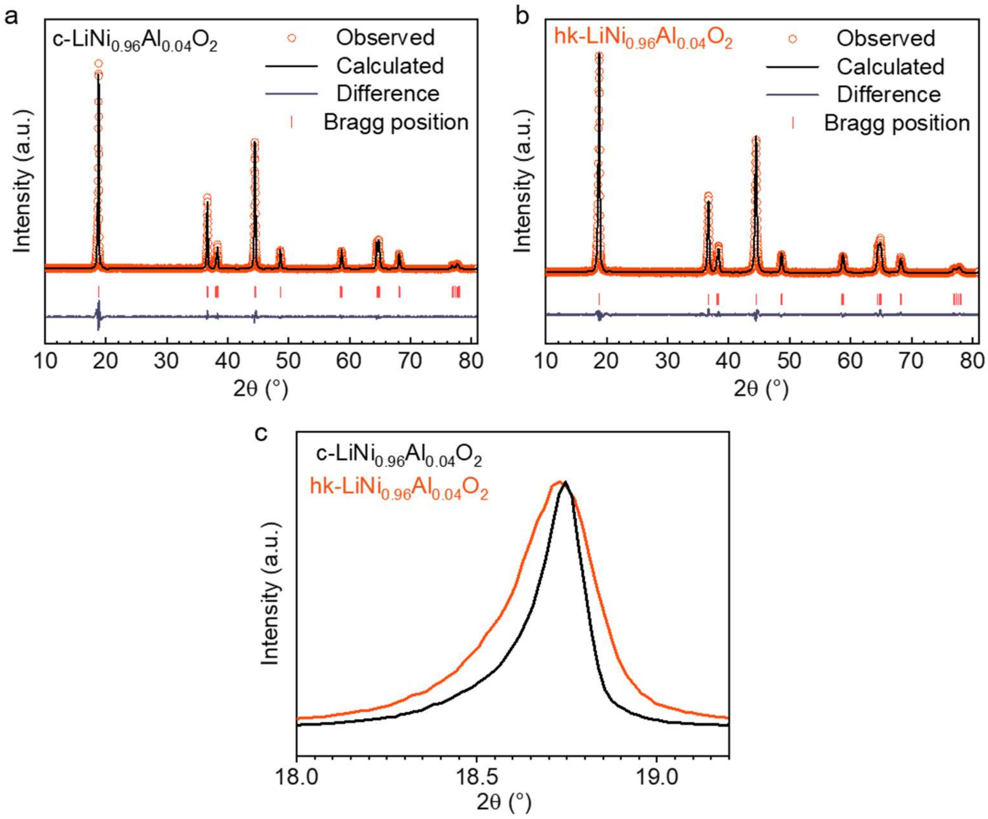

Fig. S6 a, b XRD patterns of c-$LiNi_{0.96}Al_{0.04}O_2$ and hk-$LiNi_{0.96}Al_{0.04}O_2$ including Rietveld refinement analysis, the fitting results of which are presented in **Table S2**. **c** Comparison of the (003) XRD reflection between c-$LiNi_{0.96}Al_{0.04}O_2$ and hk-$LiNi_{0.96}Al_{0.04}O_2$.

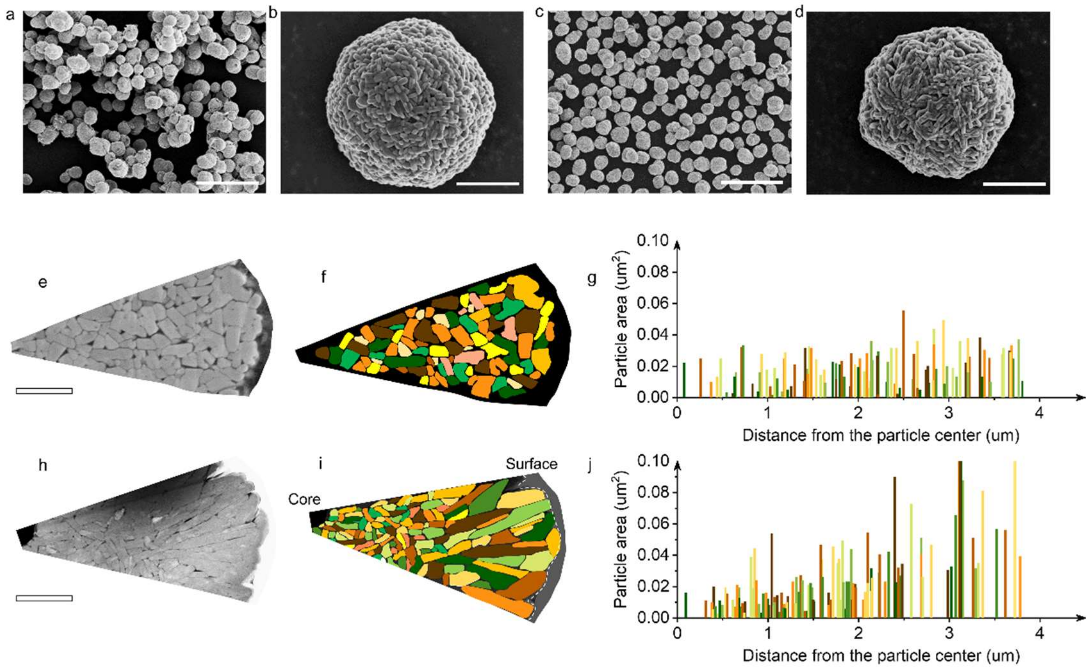

Fig. S7 Internal morphology within the secondary particle. SEM images of (**a, c**) c-$LiNi_{0.96}Al_{0.04}O_2$ and (**b, d**) hk-$LiNi_{0.96}Al_{0.04}O_2$ secondary particles with a close particle size distribution and different primary particle morphology. Cross-sectional images and the corresponding schematic illustration of the c-$LiNi_{0.96}Al_{0.04}O_2$ (**e, f**) and hk-$LiNi_{0.96}Al_{0.04}O_2$ (**h, i**). **g, j** Primary particle size distribution of the two samples, as a function of the distance from the center of secondary particle. Scale bar, 30 μm **a**, **c**; 3 μm **b**, **d**, **f**, **i**; 1 μm **e**, **f**, **h**, **i**.

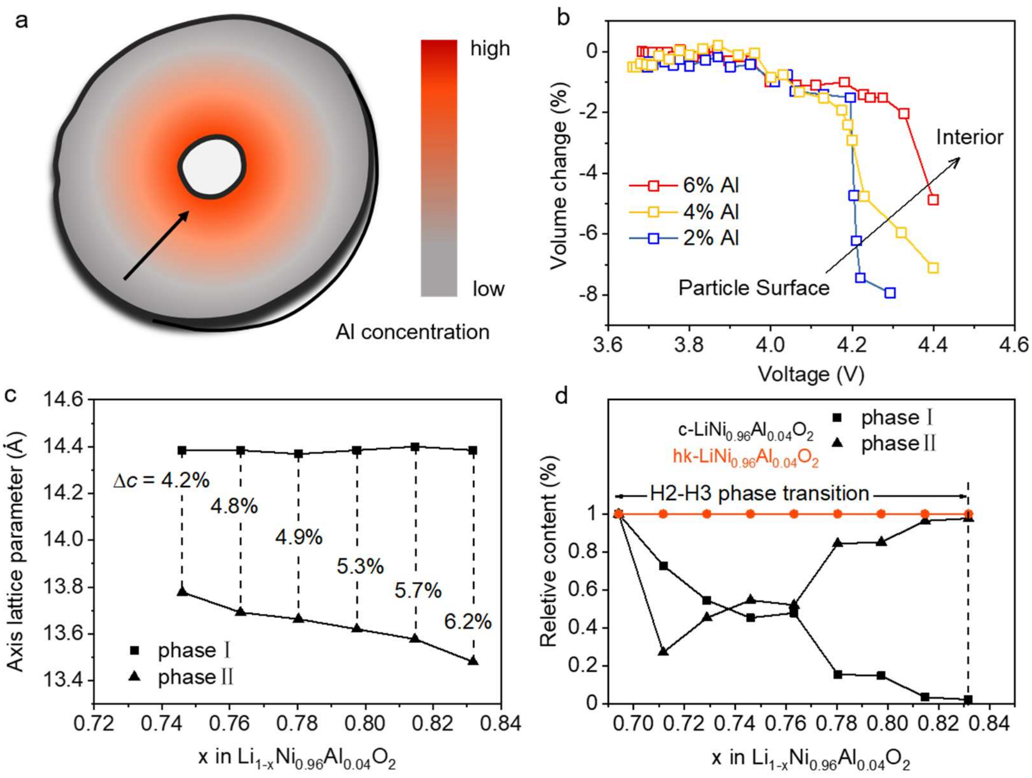

Fig. S8 Volume change and SOC heterogeneity within secondary particle. a Scheme of the resulting secondary particle with interior-rich Al dopant and central void structure. **b** Volume change of micro-lattice as a function of charge voltage under different aluminum doping amounts. **c** The evolution of the lattice parameter *c* as a function of delithiation for the two separated phase. **d** Two-phase separation within hk-$LiNi_{0.96}Al_{0.04}O_2$ compared with the control sample during H2-H3 phase transition.

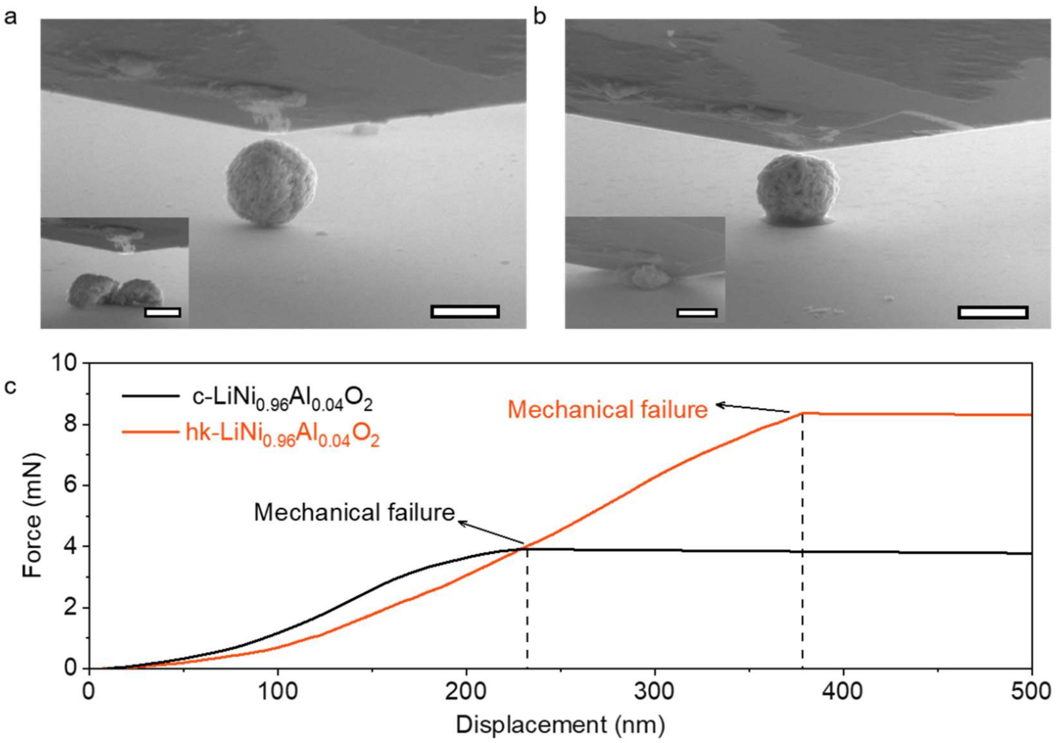

Fig. S9 The nano-indentation experiment. a, b SEM image captured the nano-indentation approach before and after mechanical failure of the representative c-$LiNi_{0.96}Al_{0.04}O_2$ and hk-$LiN_{i0.96}Al_{0.04}O_2$ particle. **c** Corresponding force-displacement curves for indentation phases of the two samples. Scale bar, 5 μm **a**, **b**.

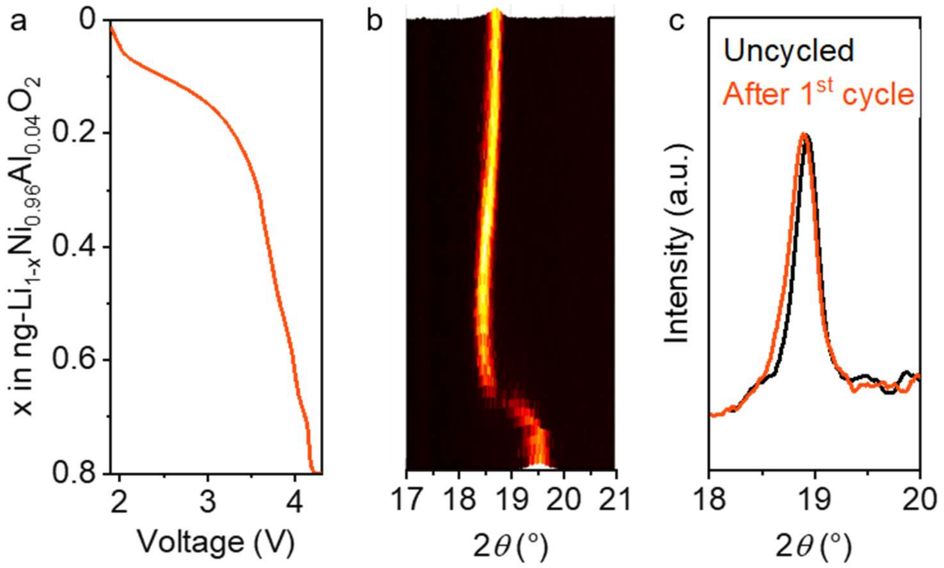

Fig. S10 Operando XRD of hk-$LiNi_{0.96}Al_{0.04}O_2$ at C/8 rate during discharge. a Voltage−delithiation depth curve of hk-$LiNi_{0.96}Al_{0.04}O_2$ during discharge and **b** corresponding contour plot of (003) reflection. **c** Magnified image of the (003) peak pattern before and after 1st cycle.

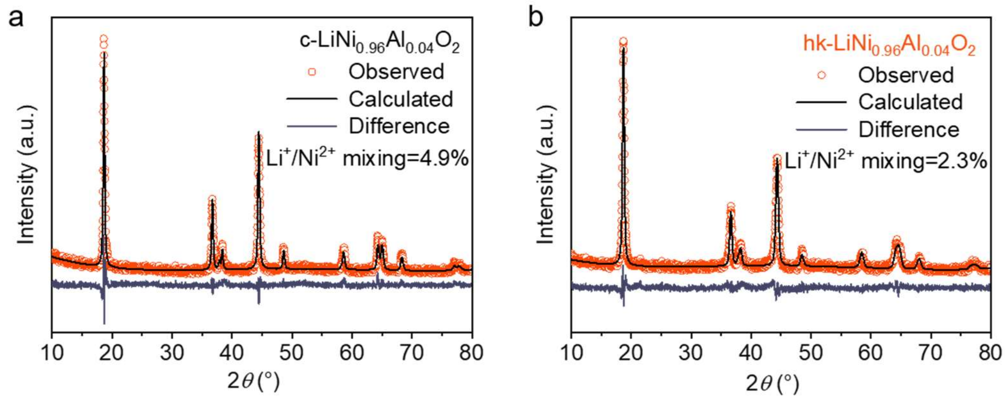

Fig. S11 XRD patterns of cycled **a** c-$LiNi_{0.96}Al_{0.04}O_2$ and **b** hk-$LiNi_{0.96}Al_{0.04}O_2$ with Rietveld refinement analysis after 500 cycles in a half-cell cycled between 2.8 and 4.4V using a constant current of C/3 (about 60mAg^{-1}) at 35 °C.

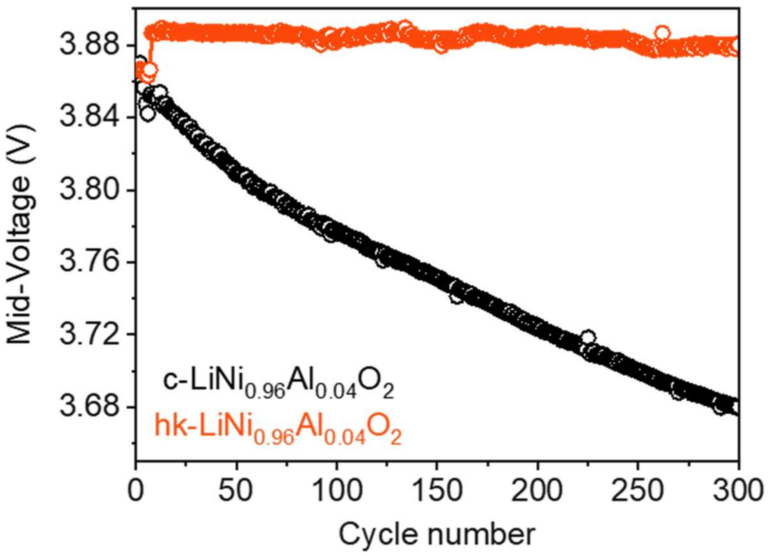

Fig. S12 Discharge mid-voltage of of c-$LiNi_{0.96}Al_{0.04}O_2$ and hk-$LiNi_{0.96}Al_{0.04}O_2$ during cycle.

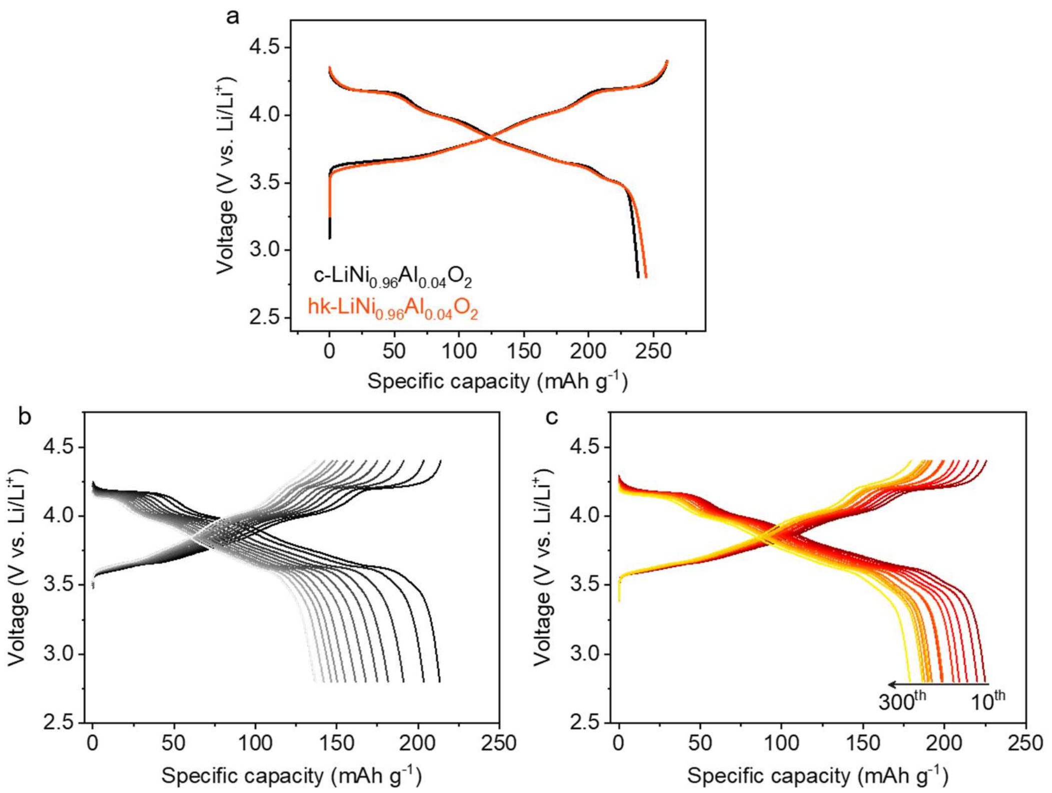

Fig. S13 a Charge/discharge profiles of c-$LiNi_{0.96}Al_{0.04}O_2$ and hk-$LiNi_{0.96}Al_{0.04}O_2$ half-cells upon the initial activation cycle between 2.8 and 4.4 V using a constant current of 0.1C (180 mA g^{-1}) at 35 °C. **b, c** Charge-discharge profiles of half-cells c-$LiNi_{0.96}Al_{0.04}O_2$ (**b**) hk-$LiNi_{0.96}Al_{0.04}O_2$ (**c**) at the 10th cycle, 25th cycle, 50th cycle until 300th cycle with an interval of 25 cycles using a constant current of 0.3C at 35 °C.

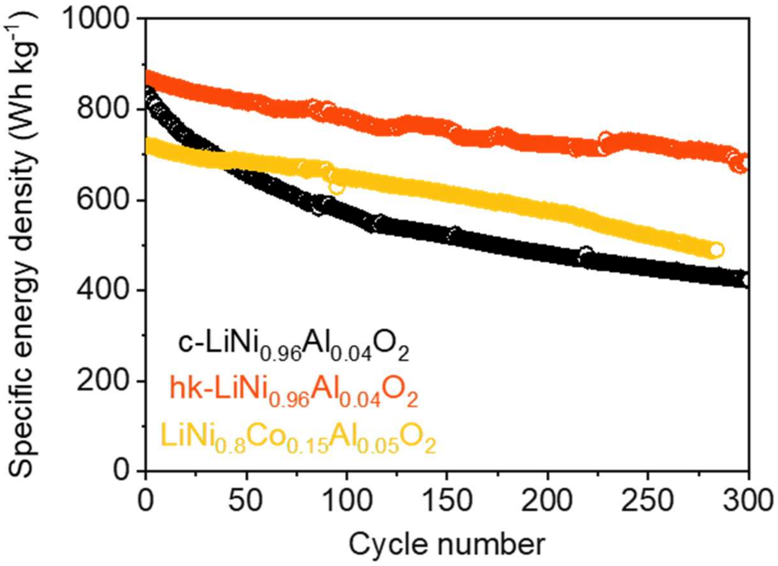

Fig. S14 Specific energy density of c-$LiNi_{0.96}Al_{0.04}O_2$, commercial $LiNi_{0.8}Co_{0.15}Al_{0.05}O_2$ and hk-$LiNi_{0.96}Al_{0.04}O_2$ during cycle.

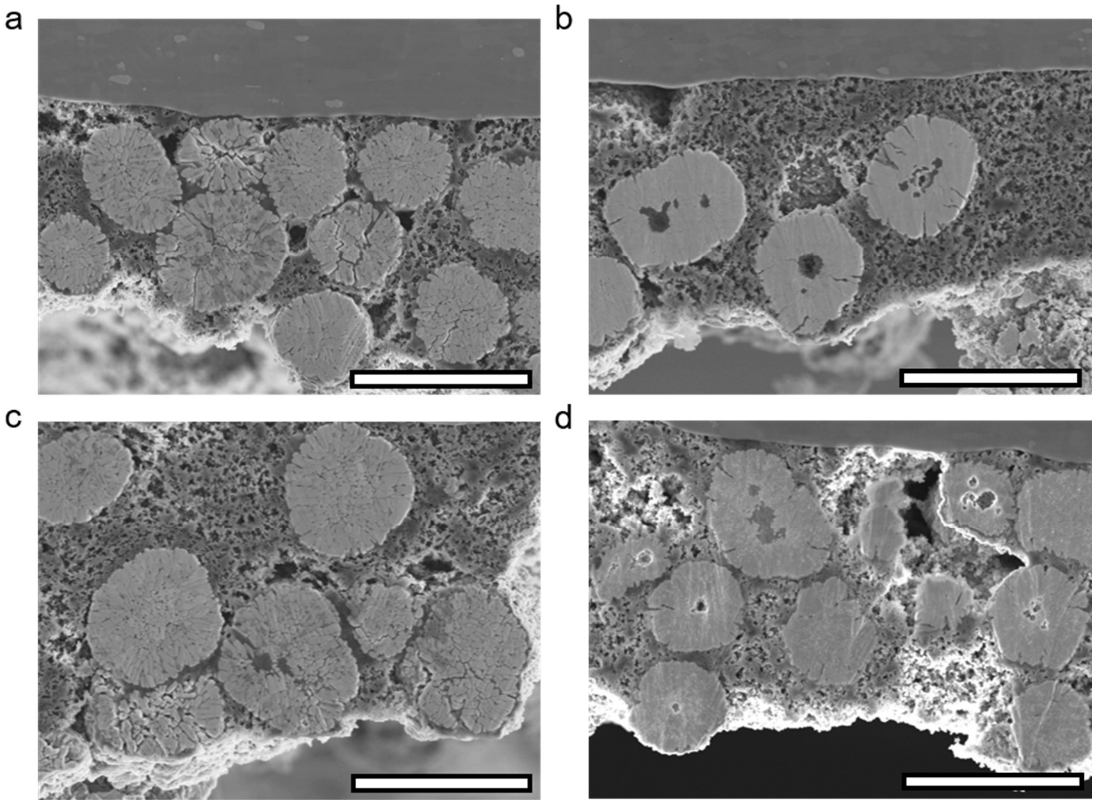

Fig. S15 Cross-sectional SEM images of **a, c** c-$LiNi_{0.96}Al_{0.04}O_2$ and **b, d** hk-$LiNi_{0.96}Al_{0.04}O_2$ in low magnification (**a, b**) after 300 cycles (**c, d**) charged to 4.6V at 1st cycle. Scale bar, 10 μm.

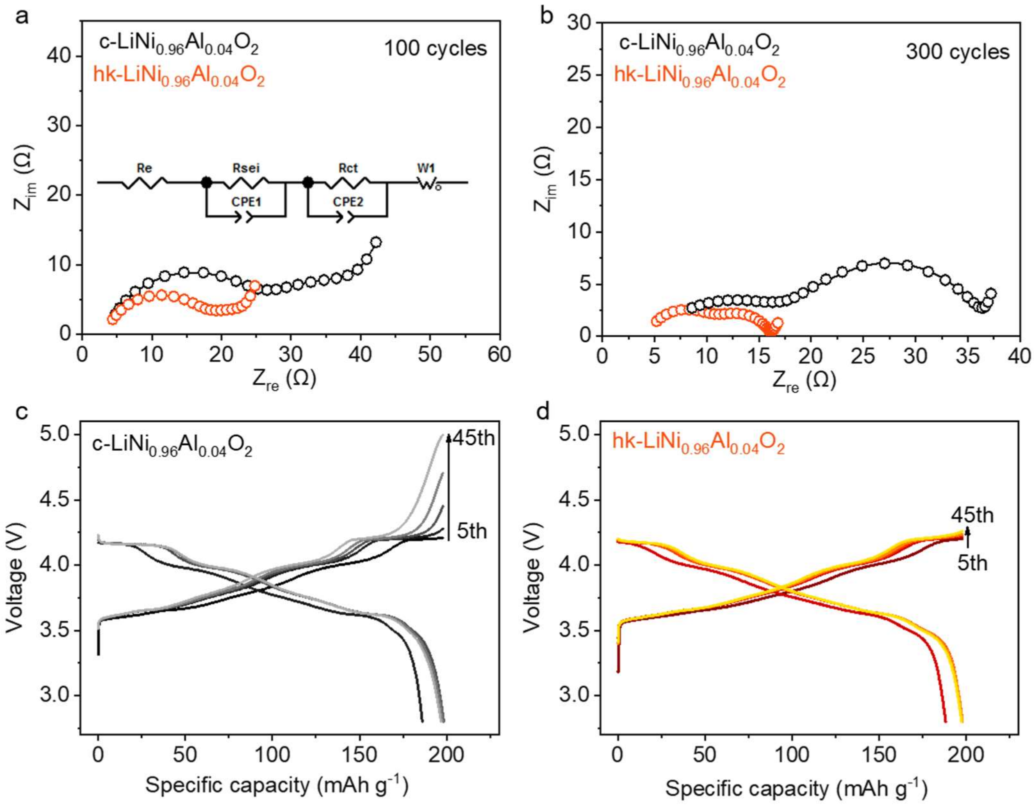

Fig. S16 Impedance analysis. a, b EIS analysis of c-$LiNi_{0.96}Al_{0.04}O_2$ and hk-$LiNi_{0.96}Al_{0.04}O_2$ after (**a**) 100 cycles (**b**) 300 cycles. **c, d** Voltage profiles of half cells (vs Li^+/Li) charging to identical charge capacity (200 mAh g^{-1}) for (**c**) c-$LiNi_{0.96}Al_{0.04}O_2$ and (**d**) hk-$LiNi_{0.96}Al_{0.04}O_2$.

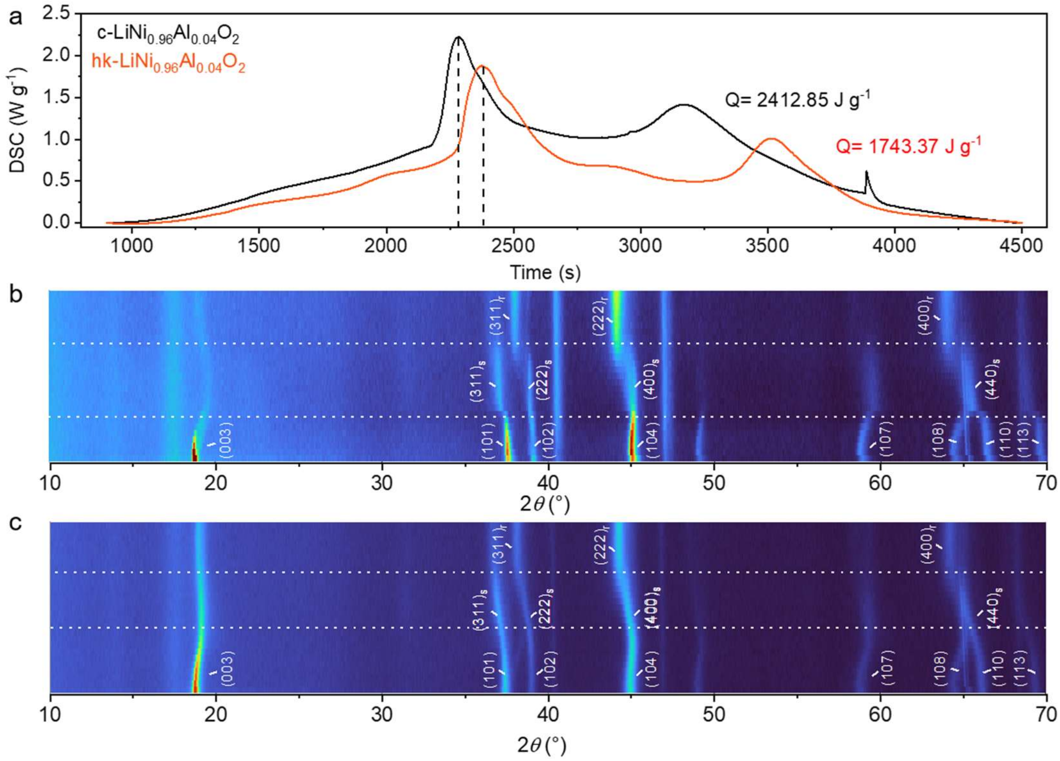

Fig. S17 Thermal stability. a DSC profile of c-$LiNi_{0.96}Al_{0.04}O_2$ and hk-$LiNi_{0.96}Al_{0.04}O_2$ (cut-off voltage at 4.3 V vs Li/Li^+) **b, c** XRD of the delithiated (cut-off at 4.3 V vs Li/Li^+) c-$LiNi_{0.96}Al_{0.04}O_2$ and hk-$LiNi_{0.96}Al_{0.04}O_2$ during in-situ heating.

DSC profiles of the two samples (**Fig. S17a**) show the higher thermal stability of hk-$LiNi_{0.96}Al_{0.04}O_2$. The c-$LiNi_{0.96}Al_{0.04}O_2$, which has the same Ni content (96%) as that of hk-$LiNi_{0.96}Al_{0.04}O_2$, delivers a notable peak temperature at 215 °C. For hk-$LiNi_{0.96}Al_{0.04}O_2$, the peak appears at 224 °C, and the enthalpy quantification of the exothermic peak is 1743.37J g^{-1}, which is much lower than that of c-$LiNi_{0.96}Al_{0.04}O_2$ (2412.85 J g^{-1}). This implies less oxygen release during the structure collapse near the material surface. During the heating, the layer-structured hk-$LiNi_{0.96}Al_{0.04}O_2$ completely transform into a spinel structure at 250 °C (**Fig. S17b, c)**, higher than c-$LiNi_{0.96}Al_{0.04}O_2$ (220°C), as evidenced by merging of the (108)R and (110)R peaks into a single peak attributed to spinel (440)S. Then both the two disordered spinel phase samples start to transform into rock salt structure ($Fm\bar{3}m$) at 320 °C. Less interfacial exposure in the charging state could explain these observations in the thermal stability test.

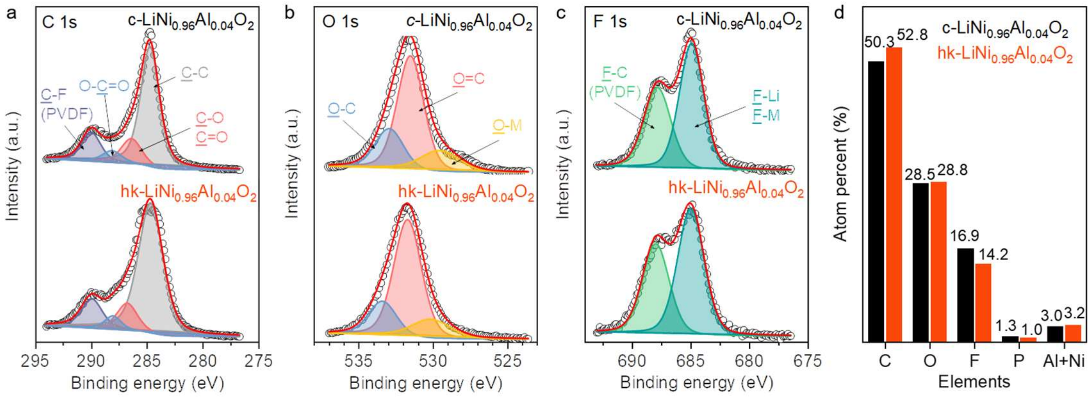

Fig. S18 XPS spectra of cycled samples. **a–c**, XPS spectra of C 1s (**a**), O 1s (**b**) and F 1s (**c**) for electrodes retrieved from half cells (vs Li/Li^+) were required without sputtering. The cells were cycled for 300 times between 2.8 and 4.4 V using a constant current of C/3 (about 60 mAg^{-1}) at 35 °C. **d** Chemical composition of the two samples surface. Atomic percent on the samples surface were obtained from XPS spectra.

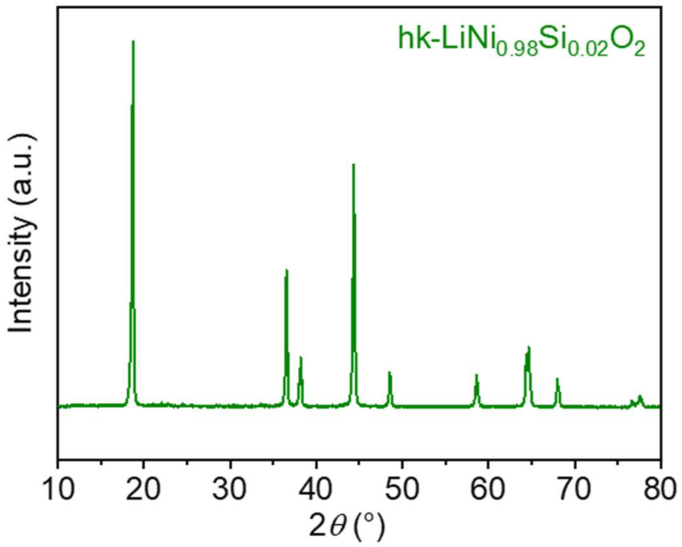

Fig. S19 XRD patterns of the precursors of hk-$LiNi_{0.98}Si_{0.02}O_2$.

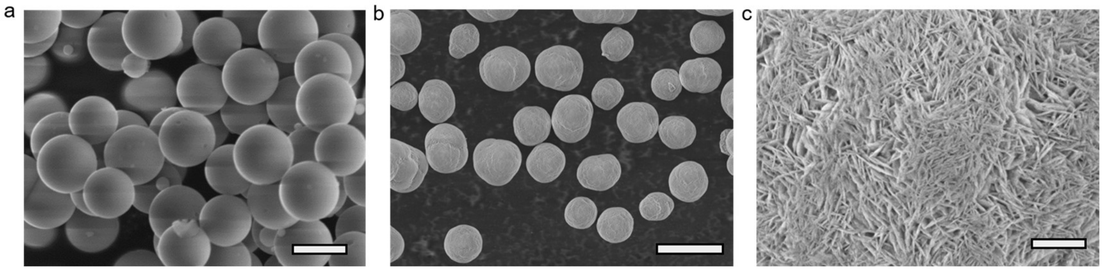

Fig. S20 Morphology of SiO_2 spherical seeds and precursor for hk-$LiNi_{0.98}Si_{0.02}O_2$. SEM images of **a** SiO_2 spherical seeds and **b, c** hk-$LiNi_{0.98}Si_{0.02}O_2$ precursors at different magnifications. Scale bar, 1 μm **a, c**; 20 μm **b**.

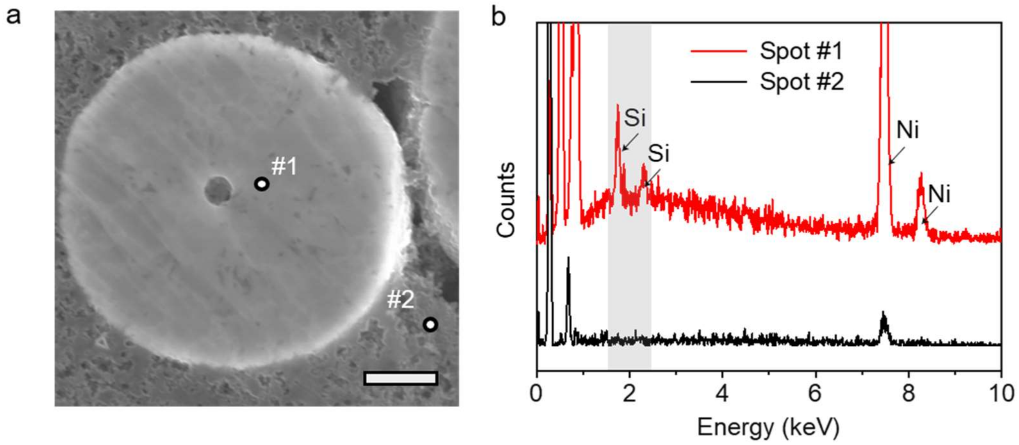

Fig. S21 a Cross-sectional SEM image of hk-$Ni_{0.98}Si_{0.02}O_2$ secondary particle. **b** EDS point-scan spectra of hk-$Ni_{0.98}Si_{0.02}O_2$ at two different positions. Scale bar, 2 μm.

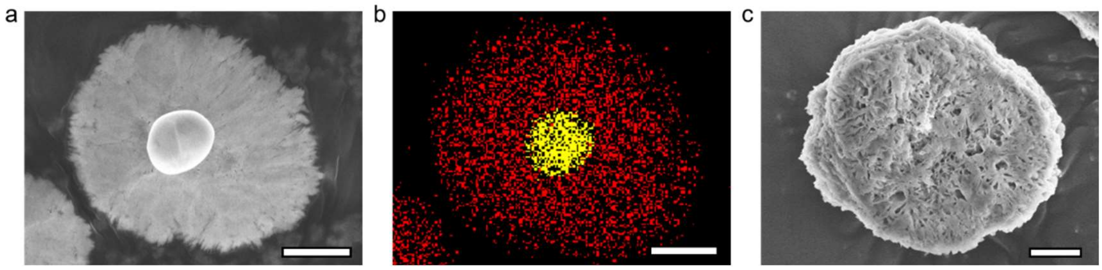

Figure S22 a Cross-sectional SEM image of the precursor for hk-$LiNi_{0.8}Co_{0.15}Al_{0.05}O_2$ cathode materials obtained by the developed co-precipitation method and **b** the corresponding element mapping of the Al_2O_3 seed within single hk-$LiNi_{0.8}Co_{0.15}Al_{0.05}O_2$ precursor. **c** Cross-sectional SEM image of the precursor for c-$LiNi_{0.8}Co_{0.15}Al_{0.05}O_2$ by classical co-precipitation method under the same synthesis conditions. Scale bar, 1 μm.

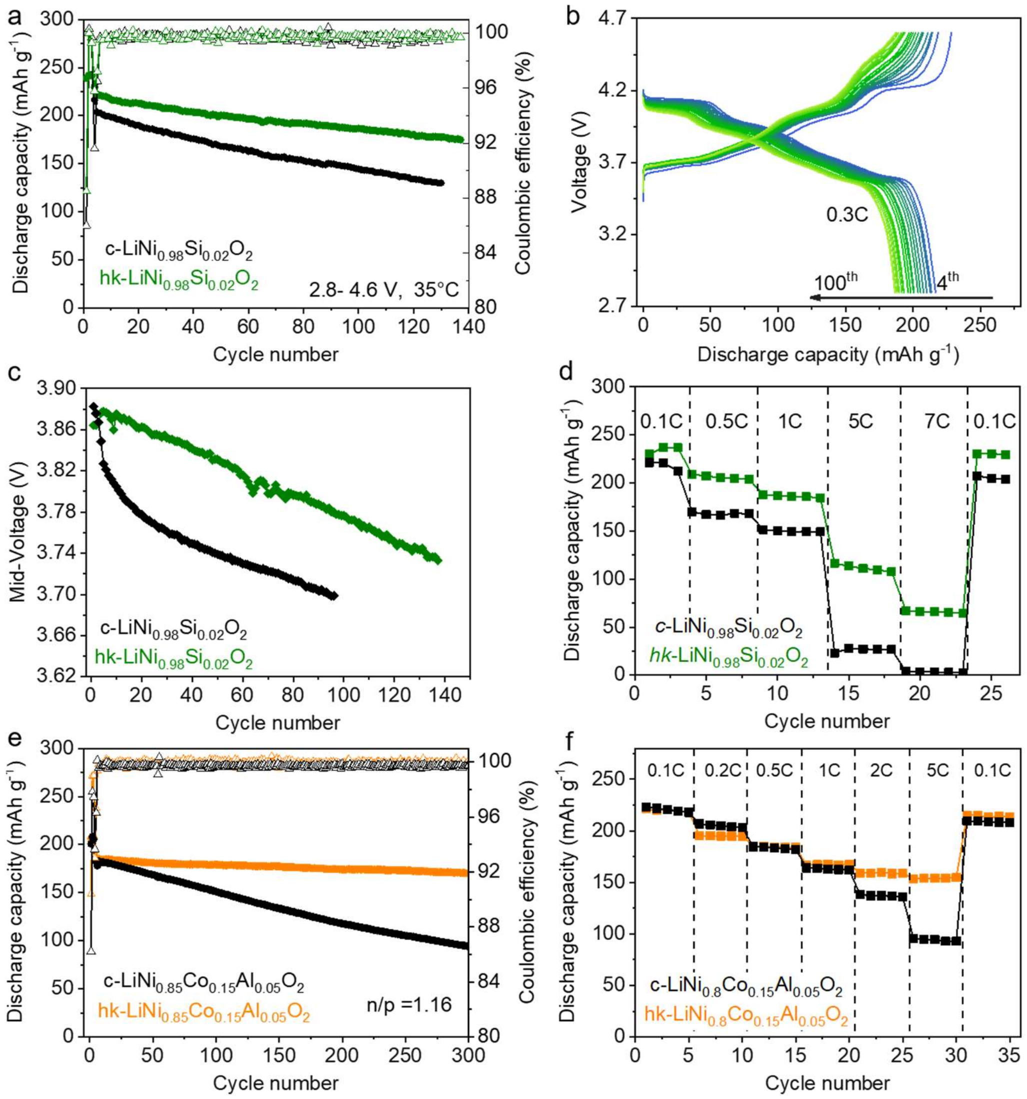

Fig. S23 Electrochemical performance of the as-obtained hk-LiNi$_{0.98}$Si$_{0.02}$O$_2$ and hk-LiNi$_{0.80}$Co$_{0.15}$Al$_{0.05}$O$_2$ cathodes. a Cycling stability of half-cells at high cut-off voltages and more practical areal loading (about 10 mg cm^{-2}) of c-LiNi$_{0.98}$Si$_{0.02}$O$_2$ and hk-LiNi$_{0.98}$Si$_{0.02}$O$_2$. **b** Charge-discharge curves of the hk-LiNi$_{0.98}$Si$_{0.02}$O$_2$ cathode at 0.3C for 100 cycles. **c** Discharge mid-voltage of half-cells for c-LiNi$_{0.98}$Si$_{0.02}$O$_2$ and hk-LiNi$_{0.98}$Si$_{0.02}$O$_2$ during cycle. **d** Rate performance of half-cells under an areal loading (about 10 mg cm^{-2}) of c-LiNi$_{0.98}$Si$_{0.02}$O$_2$ and hk-LiNi$_{0.98}$Si$_{0.02}$O$_2$ at 0.1C, 0.2C, 0.5C, 1C, 2C, 5C and 7C, respectively. **e** Cycling stability of full-cells (vs graphite anode) of c-LiNi$_{0.80}$Co$_{0.15}$Al$_{0.05}$O$_2$ and hk-LiNi$_{0.80}$Co$_{0.15}$Al$_{0.05}$O$_2$ at 0.5C. **f** Rate performance at 0.1C, 0.2C, 0.5C, 1C, 2C and 5C, respectively.

Supplementary Tables

Table S1. ICP analysis results of c-$LiNi_{0.96}Al_{0.04}O_2$ and hk-$LiNi_{0.96}Al_{0.04}O_2$.

Sample	Li (mol/L)	Ni (mol/L)	Al (mol/L)	Ni: Al
c-$LiNi_{0.96}Al_{0.04}O_2$	0.137	0.130	0.005	96.3:3.7
hk-$LiNi_{0.96}Al_{0.04}O_2$	0.153	0.148	0.006	96.1:3.9

Table S2. Rietveld refinement analysis fitted results of three samples.

Sample	a	c	c/a	$I_{(003)}/I_{(104)}$	R_{wp}	Li/Ni
$LiNiO_2$	2.8766	14.187	4.932	1.499	14.0	1.98%
c-$LiNi_{0.96}Al_{0.04}O_2$	2.8748	14.189	4.936	1.630	14.3	1.45%
hk-$LiNi_{0.96}Al_{0.04}O_2$	2.8725	14.190	4.940	1.627	10.9	2.17%

Table S3. EIS analysis results of c-$LiNi_{0.96}Al_{0.04}O_2$ and hk-$LiNi_{0.96}Al_{0.04}O_2$. The two samples were sintered with the same parameters.

Sample	R_s	R_{SEI}	R_{ct}	Chi-Squared
c-$LiNi_{0.96}Al_{0.04}O_2$	4.485	44.75	1148	0.0004
hk-$LiNi_{0.96}Al_{0.04}O_2$	4.451	45.52	1196	0.0008

Table S4. Surface residual lithium measurement.

Sample	LiOH	Li_2CO_3
c-$LiNi_{0.96}Al_{0.04}O_2$	1.80%	1.24%
hk-$LiNi_{0.96}Al_{0.04}O_2$	1.93%	1.27%

Reference

1. Jain, A. *et al.* Commentary: The Materials Project: A materials genome approach to accelerating materials innovation. *Apl Materials* **1**, 011002 (2013).
2. Shen, Y. *et al.* Insight into the Coprecipitation-Controlled Crystallization Reaction for Preparing Lithium-Layered Oxide Cathodes. *ACS Appl. Mater. Interfaces* **13**, 717-726 (2021).
3. van Bommel, A. & Dahn, J. R. Analysis of the Growth Mechanism of Coprecipitated Spherical and Dense Nickel, Manganese, and Cobalt-Containing Hydroxides in the Presence of Aqueous Ammonia. *Chem. Mater.* **21**, 1500-1503 (2009).
4. Laidler, K. J. The development of the Arrhenius equation. *Journal of Chemical Education* **61** (1984).
5. Liu, D. *et al.* Revealing the Effect of Ti Doping on Significantly Enhancing Cyclic Performance at a High Cutoff Voltage for Ni-Rich $LiNi_{0.8}Co_{0.15}Al_{0.05}O_2$ Cathode. *ACS Sustainable Chem. Eng.* **7**, 10661-10669 (2019).
6. Kong, D. F. *et al.* Ti-Gradient Doping to Stabilize Layered Surface Structure for High Performance High-Ni Oxide Cathode of Li-Ion Battery. *Adv. Energy Mater.* **9** (2019).
7. Park, G. T. *et al.* Ultrafine-grained Ni-rich layered cathode for advanced Li-ion batteries. *Energy Environ. Sci.* **14**, 6616-6626 (2021).
8. Park, G.-T. *et al.* High-performance Ni-rich $LiNi_{0.9-x}Co_{0.1}Al_xO_2$ cathodes via multi-stage microstructural tailoring from hydroxide precursor to the lithiated oxide. *Energy Environ. Sci.* **14**, 5084-5095 (2021).
